# The patient enablement instrument for back pain: reliability, content validity, construct validity and responsiveness

**DOI:** 10.1186/s12955-021-01758-0

**Published:** 2021-04-09

**Authors:** A. Molgaard Nielsen, J. Hartvigsen, A. Kongsted, B. Öberg, P. Enthoven, A. Abbott, H. H. Lauridsen

**Affiliations:** 1 Department of Sports Science and Clinical Biomechanics, University of Southern Denmark, Campusvej 55, 5230 Odense M, Denmark; 2 Chiropractic Knowledge Hub, Campusvej 55, 5230 Odense M, Denmark; 3 Department of Health, Medicine and Caring Sciences, Division of Prevention, Rehabilitation and Community Medicine, Unit of Physiotherapy, Linköping University, Linköping, Sweden

**Keywords:** Validity, Reliability, Primary care, Low back pain, Outcome assessment

## Abstract

**Background:**

Currently, there are no outcome measures assessing the ability of people with non-specific low back pain to self-manage their illness. Inspired by the ‘Patient Enablement Instrument’, we developed the Patient Enablement Instrument for Back Pain (PEI-BP). The aim of this study was to describe the development of the Patient Enablement Instrument for Back Pain (PEI-BP) and investigate content validity, construct validity, internal consistency, test–retest reliability, measurement error, responsiveness and floor and ceiling effects.

**Methods:**

The PEI-BP consists of 6 items that are rated on a 0–10 Numeric Rating Scale. Measurement properties were evaluated using the COSMIN taxonomy and were based on three cohorts from primary care with low back pain: The content validity cohort (N = 14) which participated in semi-structured interviews, the GLA:D® Back cohort (N = 272) and the test–retest cohort (N = 37) which both completed self-reported questionnaires. For construct validity and responsiveness, enablement was compared to disability (Oswestry Disability Index), back pain beliefs (Brief Illness Perception Questionnaire), fear avoidance (Fear-Avoidance Beliefs Questionnaire—physical activity), mental health (SF-36), educational level and number of previous episodes of low back pain.

**Results:**

The PEI-BP was found to have acceptable content validity, construct validity, reliability (internal consistency, test–retest reliability and measurement error) and responsiveness. The Smallest Detectable Change was 10.1 points illustrating that a patient would have to change more than 1/6 of the scale range for it to be a true change. A skewed distribution towards the high scores were found at baseline indicating a potentially problematic ceiling effect in the current population.

**Conclusions:**

The PEI-BP can be considered a valid and reliable tool to measure enablement on people seeking care for non-specific LBP. Further testing of the PEI-BP in populations with more severe LBP is recommended.

*Trial registration*: Not applicable.

**Supplementary Information:**

The online version contains supplementary material available at 10.1186/s12955-021-01758-0.

## Background

Most people experience low back pain (LBP) some time in their life [1], and most LBP is classified as non-specific because the exact nociceptive source cannot be identified with certainty [2]. Most recent guidelines for the management of non-specific LBP endorse interventions that support self-management [3]. However, currently there are no reliable ways of measuring the concept of the patients’ ability to manage their illness specific to non-specific LBP, as the most commonly used outcome measures are pain, disability and quality of life [4].

In 1997 Howie et al. presented the concept of enablement representing patients’ empowerment and ability to understand and cope with their health and illness. In order to measure enablement, they developed the ‘Patient Enablement Instrument’ (PEI) based on the theory that other important outcomes will improve if the patients experience increased enablement after a consultation in primary care [5–8]. The PEI has since been translated to multiple languages and has generally shown moderate to good validity and reliability in different settings [7, 9–17]. However, there are limitations with the use of the PEI as an outcome measure [13] including that the PEI provides a retrospective transition rating at one time-point after an intervention challenging the measurement of change over time (responsiveness) [18].

Inspired by the PEI, researchers from Denmark and Sweden created the Patient Enablement Instrument for Back Pain (PEI-BP) which could potentially be used as an outcome measure for interventions aiming to improve self-management in people who seek care for LBP. However, before using the instrument as an outcome measure in research settings or in clinical practice, further investigation of the clinimetric properties of the instrument are needed.

Therefore, the aim of this project was to investigate the validity, reliability and responsiveness of the Patient Enablement Instrument for Back Pain. The specific objectives were to describe the development of the PEI-BP and investigate content validity, construct validity, internal consistency, test–retest reliability, measurement error, responsiveness and floor and ceiling effects when applied to patients with non-specific LBP who consult either chiropractors or physiotherapists in Denmark.

## Methods

### Development of the patient enablement instrument for back pain (PEI-BP)

The PEI-BP was adapted from the original PEI [5, 7, 8] with the aim of being able to measure patients’ perceived change in ability to understand and cope with their back problem. We convened a group of experienced back pain researchers from Denmark and Sweden (JH, AK, BÖ, PE, AA) and discussed needed modifications and arrived at the following changes from the original PEI: (1) The questions in PEI-BP focus on back pain and not illness in general, (2) it enquires about the patient’s state during the past week which allow for measuring time specific changes by repeating measures of PEI-BP before and after an intervention and, (3) the responses to the 6 questions are rated on 0–10 point Numeric Rating Scales as opposed to 0–2 point scales with the aim of increasing sensitivity to change over time, i.e. responsiveness.

The PEI-BP consists of 6 items measured on a 0–10 numeric rating scale (0 = to a very low degree; 10 = to a very high degree) with a total maximum score of 60, with high scores indicating higher enablement. To make the PEI-BP available to international researchers, an English translation and cross-cultural adaptation of the PEI-BP was conducted by two independent native-English speaking persons with non-clinical backgrounds. The final translation committee also included two health professionals with one of these being an expert in the methodology. There were minor differences when comparing the translations of the PEI-BP from Danish to English, however, the translation committee reached consensus after discussions on the content of the questions. The Danish PEI-BP was used in this project and is available in Additional file 1 which also includes an English and Swedish translation. All versions of the PEI-BP are available from www.spoergeskemaer.dk/pei-bp.

### Testing of the PEI-BP

Measurement properties for the PEI-BP were evaluated based on three cohorts: The content validity cohort, the GLA:D® Back cohort and the test–retest cohort. Reporting will follow Guidelines for Reporting Reliability and Agreement Studies (GRASS) by Kottner et al. [19] and will adhere to the standardised terminology and definitions of measurement properties as described in the COSMIN (COnsensus-based Standards for the selection of health Measurement INstruments) taxonomy [18].

### The content validity cohort

From March and until May 2017, selected physiotherapy and chiropractic clinics with group-based back training were contacted by two student assistants from the University of Southern Denmark. Volunteering clinics were asked to identify five patients with back pain who were willing to participate in a semi-structured interview. The patients should participate in supervised training (individually or group-based), be at least 18 years of age and have had back pain for at least 4 weeks before the first appointment in the clinic. Consenting participants received the PEI-BP questionnaire at the clinic and were asked to complete it the same day. The clinics sent contact details from the consenting participants to the student assistants who also completed the interviews.

### The GLA:D® back cohort

The GLA:D® Back cohort was established to test the feasibility of implementing the GLA:D® Back programme in primary care chiropractic and physiotherapy clinics in Denmark [20]. Four chiropractic clinics and five physiotherapy clinics which had expressed interest in GLA:D® Back participated in the feasibility testing. Recruitment of patients was carried out between August and December 2017. Eligible patients had non-specific low back pain (LBP), were at least 18 years of age, and could speak and write Danish. There were no other firm criteria for inclusion or exclusion as clinicians decided in collaboration with the patients whether the GLA:D® Back intervention would be suitable for them. This study only included participants in the GLA:D® Back intervention arm of the feasibility study. Further details about the GLA:D® Back programme and the recruitment of clinics and patients have been reported previously [20–22].

A target sample size of at least 200 participants was planned for this cohort which was estimated to ensure a stable variance–covariance matrix in an exploratory factor analysis. A sample size of 4–10 participants per item and more than 100 subjects has been suggested as sufficient [23].

### The test–retest cohort

The test–retest cohort was established specifically for this study by two student assistants at the University of Southern Denmark as data for analysing test–retest was not available from the GLA:D*®* Back Cohort. Participants were recruited from two primary care physiotherapy and three chiropractic clinics in the region of Southern Denmark between September and November 2018. Inclusion criteria were established to recruit a comparable cohort based on the characteristics of the GLA:D® Back cohort: (1) at least 18 years of age, (2) LBP had impacted activities of daily living for more than 1 month, (3) at least 3 consultations for treatment within the past 2 years, (4) no pain below the knee and (5) could speak and understand Danish.

As the overall leg pain intensity of the GLA:D*®* Back Cohort was low, we deemed that including patients with ‘no pain below the knee’ was the best way of getting a comparable cohort.

For a valid test of reliability, at least 50 participants were expected to complete both questionnaires and with no change in back pain intensity between the two time points [24]. The study was advertised in the clinics and the secretaries were asked to help recruiting patients fulfilling the inclusion criteria. Interested patients signed an electronic consent form.

### Data collection

*In the content validity cohort* semi-structured telephone interviews were conducted and audio recorded. The interviews included two main topics: (1) comprehensibility of each item of the questionnaire, and (2) the overall relevance of the questionnaire and response options. For each item, the respondents were asked about their interpretation of the item. Responses were compared to pre-defined response themes including “I don’t know” and responses unrelated to these options were noted. The themes covered the types of responses that had been considered most likely for each item beforehand. The participants were also encouraged to add any comments they found relevant during the interview. Recruitment continued until the same responses were obtained, and during four subsequent interviews no new themes emerged.

*In the GLA:D® Back and the test–retest cohorts*, participants completed self-reported questionnaires which covered demographics, patient back pain history and core outcome domains for the evaluation of back pain in clinical trials according to the consensus statement by Chiarotto et al. [4]. Additionally, more specific instruments measuring aspects related to the patients’ abilities of self-management were included. Details about the PEI-BP are described above, and further details of the remaining content of the self-reported questionnaires are reported in Table 1.

**Table 1:** Description of the baseline characteristics and outcome variables collected in the three cohorts of participants

	Content validity cohort	GLA:D® Back cohort	Test–retest cohort
**Baseline characteristics**			
Age, years	✓	✓	✓
Sex, male/female	✓	✓	✓
Education: no qualification; public school; high school; vocational training; higher education < 3 years; higher education 3–4 years; higher education > 4 years	✓	✓	✓
Back pain intensity: Numeric rating scale 0–10 (0 = no pain, 10 = worst imaginable pain) [25]	✓	✓	✓
Leg pain intensity: Numeric rating scale 0–10 (0 = no pain, 10 = worst imaginable pain) [25]	✓	✓	✓
Episode duration (5 -point scale): 0–2 weeks, 2–4 weeks, 4–12 weeks, 3–12 months, > 1 year	✓	✓	✓
Previous back pain episodes (4-point scale): 0 episodes, 1 episode, 2–3 episodes, > 3 episodes	✓	✓	✓
STarT Back Tool (SBT): Contains 9 items each with a score of 0 or 1 with a higher score indicating higher risk of poor prognosis. Risk groups are based on the total score and a sub score (Q5-9): Low risk (3 or less on the total score), medium risk (4 or more on total score and 3 or less on sub score) and high risk (4 or more on both total score and sub score) [26, 27]		✓	✓
**Outcome measures**			
Pain-related disability			
Oswestry Disability Index (ODI): Contains 10 items (pain intensity, personal care, lifting, walking, sitting, standing, sleeping, sex life, social life, traveling) each with 6 response options on a 0–5 point scale. The answers on the 10 items are converted into a single score (0–100), higher scores indicate more disability [28–30]		✓	✓
Mental health			
Brief Illness Perception Questionnaire (BIPQ): Contains 9 items each assessing one dimension of illness perception (consequences, timeline, personal control, treatment control, identity, illness concern, coherence, emotional representation and a causal item). Item 1–8 are scored on a 11-point scale (0–10) and converted to a sum score (range 0–80), higher scores reflects more threatening view of the back pain [31, 32] For analysis of single items (3 and 7) the response categories were reversed, and a lower score reflected a more threatening view of the back pain		✓	
Fear-Avoidance Beliefs Questionnaire (FABQ) – physical activity subscale: Contains 5 items with Likert response options scored on a 0–6 point scale (0 = Completely disagree, 6 = completely agree) of which item 2–5 are included in the score (range 0–24). Higher scores indicate higher fear avoidance beliefs [33, 34]		✓	
SF-36 subscale ‘mental health’: Contains 5 items on a 1–6 point scale (1 = All of the time, 6 = None of the time), which are converted into a single score (0–100). Higher scores indicate a more favorable mental health [35, 36]		✓	
Quality of life			
SF-36 subscale ‘social functioning limited by physical health’: Contains 1 item on a 1–6 point scale (1 = All of the time, 0 = None of the time). Higher scores indicate a more favorable social functioning [35, 36]		✓	

SF-36 = Short Form 36, version 1.0

*In the GLA:D® Back cohort*, the questionnaire was sent electronically using the REDCap software provided and supported by the Open Patient data Explorative Network (OPEN) [37]. Participants received an e-mail with a link to the questionnaires on the day of the baseline consultation and 4 months later. If no response within 3 days, a reminder was sent.

*In the test–retest cohort,* the questionnaires were sent electronically using the survey-tool SurveyXact provided and supported by Ramboll [38]. Consenting participants received an e-mail asking them to complete the online baseline and follow-up questionnaires with 3–5 days in between. If no response within 2 days, a reminder was sent. If still no response within another couple of days, the participant was reminded using a phone call.

### Statistical analysis

In the descriptive analysis, nominal scale variables were presented as proportions and continuous scale variables as mean and standard deviation. Baseline comparisons within the GLA:D® Back cohort between the participants who completed the baseline and follow-up questionnaires and the ones who only completed the baseline questionnaire were tested using χ^2^ test for nominal variables and Mann–Whitney U test for continuous variables. Using the same test strategy, baseline comparisons were performed between the GLA:D® Back cohort and the test–retest cohort. A p-value < 0.05 was considered significant. If any of the 6 included items in the total score of PEI-BP was missing at baseline or follow-up, no sum-score was calculated, and the scale was discarded. Measurement properties of the PEI-BP were evaluated using the COSMIN taxonomy [24].

#### Item analyses

The 6 items of the PEI-BP were examined through item distribution, kurtosis and skewness. If less than 3% were missing for each item, this was considered acceptable [24].

#### Content validity

The interviews were analysed quantitatively by assessing, for each item, how many respondents had a similar interpretation of the question, that is if they chose the same pre-defined response theme. Further, it was summarised how many respondents considered the overall questionnaire easy to complete and relevant. Further comments from the participants during the interview were additionally taking into consideration when evaluating the overall content validity of the questionnaire.

#### Construct validity

##### Structural validity

An explorative factor analysis (EFA) was conducted to identify the underlying factor structure of the PEI-BP. Initially, sampling adequacy was tested using Bartlett’s Test of Sphericity and the Kaiser–Meyer–Olkin test [39]. The EFA was carried out using a principal axis factor analysis with a polychoric correlation matrix combined with an oblique oblimin rotation and Kaiser normalisation to obtain meaningful and correlated factors if present [40]. The number of factors was examined using a scree plot, and factors with eigenvalues of > 1 were retained [24]. Factor loadings of > 0.7 and communalities of > 0.5 were considered satisfactory [39].

##### Hypothesis-testing

This was assessed by formulating eight hypotheses regarding the size and direction of correlations. The hypotheses were between the PEI-BP summary score and selected baseline questions (Table 5), four instruments (BIPQ, ODI, FABQ, SF-36) and one individual item (BIPQ-3:*“how much control do you feel you have over your back pain”)*. One additional hypothesis was formulated regarding the size and direction of the correlation between the second PEI-BP item (“*to which degree were you able to understand your back problem”*) and the seventh BIPQ item (*“how well do you feel you understand your back pain”*). The relationships between the PEI-BP and other measurements were investigated using Spearman’s rank correlation. The strength of the correlations was formulated according to Cohen’s criteria (low ≤ 0.1, moderate > 0.1; ≤ 0.3 and high > 0.5) [41]. As an indicator of the strength of evidence for construct validity of the PEI-BP, the percentage of correctly predicted hypotheses were determined [24, 42].

#### Reliability

Internal consistency was determined from baseline data in the GLA:D® Back cohort study using Cronbach’s α. Alpha was established after completion of the factor analysis and was considered satisfactory between 0.7 and 0.9 [24].

Data from the test–retest cohort was used to assess the test–retest reliability using an Intra Class Correlation with 95% confident intervals (CI) based on a two-way mixed-effects model with single rater/measurement and absolute agreement (equivalent to an ICC(2.1)A). A value ≥ 0.70 was considered acceptable [24, 43].

Measurement error was assessed using Bland and Altman plots [44]. The smallest detectable change (SDC) was defined as change outside the limits of agreement [45].

#### Responsiveness

Responsiveness was investigated using data from the GLA:D® Back cohort. Construct responsiveness was assessed by formulating six hypotheses regarding the size and direction of correlations between the PEI-BP change score and change scores of selected instruments (BIPQ, ODI, FABQ, SF-36) and the third BIPQ item [24, 46]. One additional hypothesis was formulated regarding the size and direction of the correlation between the change score of the second PEI-BP item and the seventh BIPQ item (Table 5).

#### Floor and ceiling effects

Floor and ceiling effects were assessed using both the classical method [47] and the”scale width” method [48]. The latter is defined as the capacity of a scale to have baseline scores far enough onto the scale (the smallest detectable change) to allow detection of change in scores over time [48]. Scale width was considered acceptable if no more than 15% of the subjects had baseline PEI sum scores falling outside the scale width either at the upper or lower end of the scale.

Statistical analyses were conducted using STATA/IC 16.1 (StataCorp LP, College Station, TX, USA).

## Results

### Participant characteristics

#### The GLA:D® Back cohort

**Fig. 1:**
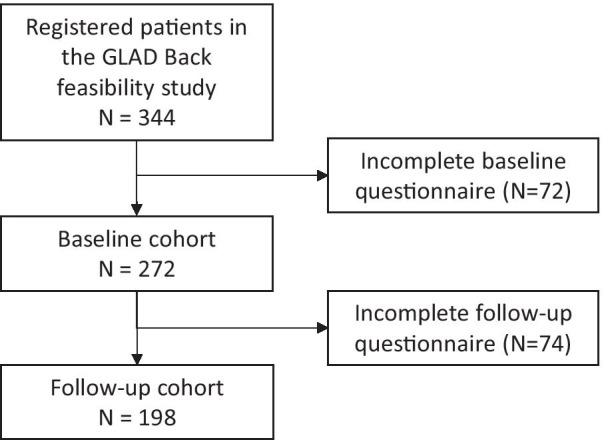
The GLAD® Back cohort used for clinicmetric analyses of the Patient Enablement Instrument for Back Pain

For the GLA:D® Back cohort, a total of 272 (79%) and 198 (58%) participants responded to the baseline and follow-up questionnaires, respectively (Fig. 1). Comparison of baseline characteristics revealed no significant statistical differences between the participants who completed both questionnaires and the participants who only completed the baseline questionnaire when tested for age, sex, back pain intensity, episode duration, PEI-BP sum score and ODI sum score. The mean PEI-BP sum score increased from 41.8 (SD 10.8) at baseline to 48.2 (SD 10.0) at follow-up. Further baseline characteristics are available in Table 2.

**Table 2:** Patient reported baseline characteristics for the cohorts of participants with non-specific low back pain

	Content validity cohort N = 14	GLA:D® Back baseline cohort N = 272	Test–retest cohort N = 37
Age, mean (SD, years)	56 (8)	53 (12)	52 (13)
Males, N (%)	3 (21)	57 (21)	13 (35)
Highest achieved education, N (%)			
No qualification	0	1 (0.4)	2 (5)
Public school	0	14 (5)	0 (0)
High school	0	9 (3)	0 (0)
Vocational training	10	75 (28)	7 (19)
Higher education < 3 years	1	37 (14)	3 (8)
Higher education 3–4 years	2	100 (37)	18 (49)
Higher education > 4 years	1	26 (10)	6 (16)
Missing	0	10 (4)	1 (3)
Back pain intensity (0–10 Numeric Rating Scale), mean (SD)	4.1 (2.1)	5.0 (2.3)	5.9 (2.4)
Missing (%)	0	1 (0.4)	0
Leg pain intensity (0–10 Numeric Rating Scale), mean (SD)	7 (5.0)*	2.8 (2.7)	3.1 (2.7)
Missing (%)	0 (0)	0 (0)	0 (0)
Episode duration, N (%)			
0–2 weeks	0	18 (7)	< 1 week = 5 (14)
2–4 weeks	0	11 (4)	1–4 weeks = 4 (11)
4–12 weeks	2	18 (7)	5 (14)
3–12 months	1	52 (19)	6 (16)
> 1 year	11	172 (63)	17 (46)
Missing	0	1 (0.4)	0 (0)
Number of previous episodes, N (%)			
0 episodes	1	67 (25)	3 (8)
1 episode	1	48 (18)	3 (8)
2–3 episodes	0	53 (19)	6 (16)
> 3 episodes	12	103 (38)	25 (68)
Missing	0	1 (0.4)	0 (0)
Patient Enablement Instrument for back pain (0–60), mean (SD)		41.8 (10.8)	39.6 (13.3)
Missing, N (%) [any of 6 items missing]		11 (4)	0 (0)
STarT Back Tool score, N (%)			
Low risk [any of 9 items missing]		156 (57)	19 (51)
Medium risk		68 (25)	8 (22)
High risk		48 (18)	8 (22)
Missing (items)		7 (3)	2 (5)
Oswestry Disability Index (0–100), mean (SD)		22.5 (11.6)	22.4 (15.6)
Missing, N (%) [≥ 4 of 10 items missing]		0 (0)	0 (0)
Brief Illness Perception Questionnaire (0–80), mean (SD)		40.7 (11.0)	
Missing, N (%) [≥ 3 of 8 items missing]		2 (1)	
Fear Avoidance Belief Questionnaire—physical activity (0–24), mean (SD)		8.3 (5.4)	
Missing N (%) (any of 4 items missing)		7 (3)	
SF-36 subscales			
Mental health (0–100), mean (SD)		72.4 (16.9)	
Missing, N (%) [any of 5 items missing]		6 (2)	
Social functioning limited by physical health		1.8 (0.9)	
Missing, N (%)		0 (0)	

SF-36 = Short Form 36

^*^Leg pain yes/no, N (%)

#### The test–retest cohort

**Fig. 2:**
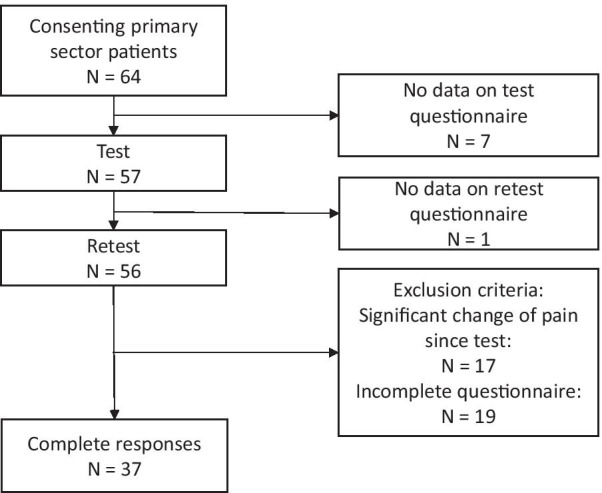
The test–retest cohort used for reliability analyses of the Patient Enablement Instrument for Back Pain

For the test–retest reliability study, 37 participants with non-specific LBP were included, who had complete responses on the PEI-BP and no significant change in pain between test and retest (Fig. 2). The response rate at retest was 37/64 (58%) and the change of the PEI-BP sum score from test to retest was 3.1 (from 39.6 to 42.7). Further baseline characteristics are available in Table 2.

Reasons for non-completion were unknown in both cohorts. Comparison of baseline characteristics revealed no significant statistical differences between the test–retest cohort and the GLA:D® Back cohort when tested for age, sex, PEI-BP sum score and ODI sum score.

#### The content validity cohort

Five clinics were contacted and three clinics recruited eight, five and three participants, respectively. Two interviews were not transcribed as saturation had been met. Baseline characteristics for the participants included in the analysis are available in Table 2.

### Item analyses

#### GLA:D® back cohort

The distribution of baseline PEI-BP scores for the GLA:D® Back cohort are presented in Table 3. Generally, the entire score range was used, however the scores were skewed toward the high scores. Item 6 (*Manage your life independently*) was skewed the most toward the high scores followed by item 1 (*Handle your everyday life*). The items with the most even distribution were item 4 (*Keep your back in good health*) and item 5 (*Feel confident with your health*).

**Table 3:** Distribution of the Patient Enablement Instrument for Back Pain (PEI-BP) scores at baseline based on 272 patients with non-specific low back pain (the GLA:D® Back cohort)

Item	Response categories, N (%)											Missing N (%)	Skewness*	Kurtosis^#^
	To a very low degree										To a very high degree			
	0	1	2	3	4	5	6	7	8	9	10			
Q1: Handle your everyday life	1 (0.4)	0 (0.0)	0 (0.0)	7 (2.6)	5 (1.9)	32 (11.8)	24 (8.8)	39 (14.3)	56 (20.6)	52 (19.1)	55 (20.2)	1 (0.4)	−0.7	3.2
Q2: Understand your back problem	6 (2.2)	4 (1.5)	10 (3.7)	11 (4.0)	17 (6.3)	25 (9.2)	26 (9.6)	36 (13.2)	44 (16.2)	45 (16.5)	43 (15.8)	5 (1.8)	−0.8	2.9
Q3: Manage your back problem	0 (0.0)	1 (0.4)	11 (4.0)	12 (4.4)	16 (5.9)	29 (10.7)	34 (12.5)	41 (15.1)	50 (18.4)	41 (15.1)	32 (11.8)	5 (1.8)	−0.5	2.5
Q4: Keep your back in good health	6 (2.2)	7 (2.6)	18 (6.6)	20 (7.4)	28 (10.3)	52 (19.1)	33 (12.1)	31 (11.4)	34 (12.5)	25 (9.2)	12 (4.4)	6 (2.2)	−0.2	2.4
Q5: Feel confident with your health	5 (1.8)	7 (2.6)	26 (9.6)	18 (6.6)	20 (7.4)	42 (15.4)	22 (8.1)	25 (9.2)	41 (15.1)	35 (12.9)	27 (9.9)	4 (1.5)	−0.3	2.0
Q6: Manage your life independently	0 (0.0)	2 (0.7)	1 (0.4)	3 (1.1)	7 (2.6)	11 (4.0)	7 (2.6)	20 (7.4)	39 (14.3)	63 (23.2)	117 (43.0)	2 (0.7)	−1.7	5.9

^*^A measure of the degree and direction of asymmetry of a distribution. A symmetric (normal) distribution has a skewness of 0, and a distribution skewed to the left has a negative coefficient (e.g. when the median is greater than the mean)

^#^A measure of tailedness of a distribution. A normal distribution has a coefficient of kurtosis of 3, the smaller the coefficient of kurtosis, the flatter the distribution

Overall, there were few missing items with item 4 (*keep your back in good health*) having the highest amount of missing answers (2.2%) and item 1 (*handle your everyday life*) the lowest amount (0.4%) of missing responses (Table 3).

### Content validity

#### Content validity cohort

After fourteen semi-structured interviews, the majority of participants chose the same pre-defined response theme. Item 3 *(manage your back problem)* showed the largest dispersion while item 1 *(handle your everyday life)* and item 6 *(manage your life independently)* showed the smallest among the selected response categories.

The participants expressed uncertainty of the comprehension of item 3 and 5 participants found it difficult to distinguish item 3 from one of the other items (item 1, 2 and 4, respectively). Eleven of the participants generally found the questionnaire relevant, whereas the remaining three participants felt they had their back pain under control and considered the questionnaire as irrelevant. Relevance was mostly perceived in two ways, either as relevant in research settings or as relevant at an individual level. Overall, none of the items were deemed as unnecessary. The participants found the instrument adequately representing enablement and considered the scale range as appropriate.

### Construct validity

#### Structural validity

**Fig. 3:**
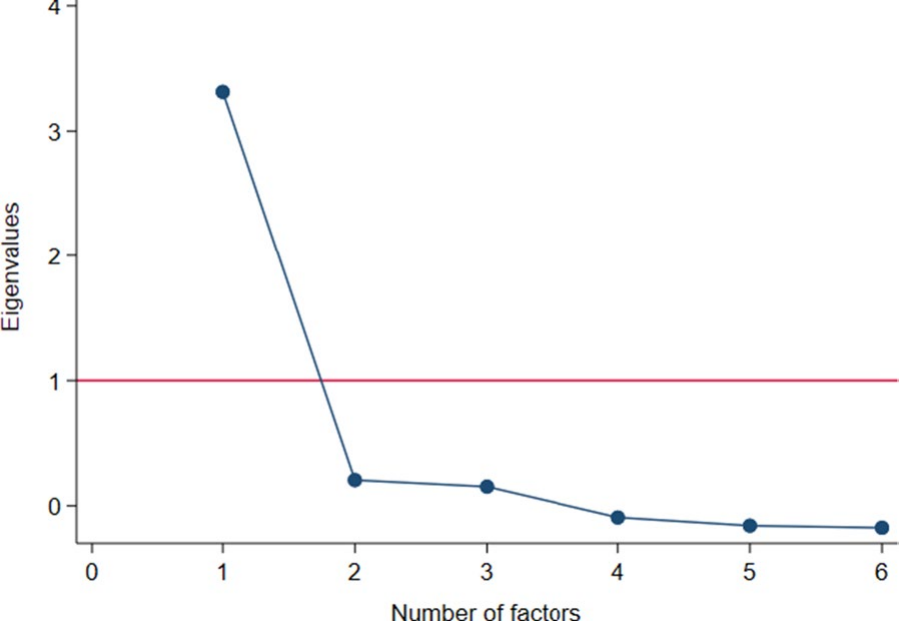
Scree plot of the Patient Enablement Instrument for Back Pain based on baseline scores from 261 patients with non-specific low back pain (The GLA:D® Back cohort)

The test for sampling adequacy showed that the sample was factorable (Bartlett’s test: *P* < 0.001; KMO = 0.84). The EFA revealed that the PEI-BP had a clear 1-factor structure (eigenvalue > 1) (Fig. 3), and this was the only model analysed. The 1-factor model showed acceptable factor loadings ranging from 0.67 to 0.83 (Table 4). Item 2 and item 6 < 50% variance explained by the identified factor whereas the remaining 4 items showed communalities ranging between 0.52 and 0.70 (Table 4).

**Table 4:** Factor structure of the Patient Enablement Instrument for Back Pain (PEI-BP) based on 261 patients with non-specific low back pain

Item	Factor 1	Communalities
Q1: Handle your everyday life	0.76	0.59
Q2: Understand your back problem	0.70	0.48
Q3: Manage your back problem	0.83	0.70
Q4: Keep your back in good health	0.73	0.52
Q5: Feel confident with your health	0.76	0.56
Q6: Manage your life independently	0.67	0.46

#### Hypothesis testing

The proportion of correctly predicted hypotheses was 88% (7/8) which indicates that the PEI-BP seem to test the intended construct (Table 5).

**Table 5:** Hypothesis testing in construct validity and construct responsiveness of the Patient Enablement Instrument of Back Pain

		Construct validity			Construct responsiveness		
			Correlations^#^			Correlations	
		Hypothesis*	Expected	Observed	Hypothesis	Expected	Observed
ODI_sum. score_	PEI-BP_sum. score_	a	< − 0.5	**− 0.5407**	a	< − 0.5	**− 0.5552**
BIPQ_sum. score_	PEI-BP_sum. score_	a	< − 0.5	**− 0.5808**	a	< − 0.5	**− 0.5375**
BIPQ_q.3_	PEI-BP_sum. score_	b	> 0.5	0.4341	b	> 0.5	**0.5484**
BIPQ_q.7_	PEI-BP_q. 2_	b	> 0.5	**0.5962**	b	> 0.5	0.4635
FABQ_phys. activity_	PEI-BP_sum. score_	c	− 0.3 to − 0.5	**− 0.3043**	c	− 0.3 to − 0.5	**− 0.4301**
SF-36 Mental health	PEI-BP_sum. score_	d	0.3 to 0.5	**0.4986**	d	0.3 to 0.5	**0.4069**
Educational level	PEI-BP_sum. score_	e	≤ 0.3	**0.1259**			
Number of previous episodes of LBP	PEI-BP_sum. score_	f	≥ − 0.3	**− 0.0365**			

ODI = Oswestry Disability Index; sum. score = summary score; PEI-BP = Patient Enablement Instrument for Back Pain; BIPQ = Brief Illness Perception Questionnaire; q = question; FABQ_phys.activity_ = Fear Avoidance Belief Questionnaire, the physical activity sub scale; SF-36 = Short Form 36

^*^Hypothesis: (a) Scales are expected to measure the same construct. The correlation is expected to be negative and high (< -0.5). (b) Scales are expected to measure the same construct. The correlation is expected to be positive and high (> 0.5). (c) Scales are related but do not measure the same construct. The correlation is expected to be negative and moderate (-0.3 to -0.5). (d) Scales are related but do not measure the same construct. The correlation is expected to be positive and moderate (0.3 to 0.5). (e) Scales are expected not to measure the same construct. The correlation is expected to be positive and low (≤ 0.3). (f) Scales are expected not to measure the same construct. The correlation is expected to be negative and low (≥ -0.3)

Bold numbers are positive hypotheses and plain numbers are negative hypotheses

^#^Spearman’s rank correlation coefficient

There was a high correlation between higher patient enablement (PEI-BP) and lower disability (ODI) (ρ = −0.54) and a lower degree of back pain beliefs (BIPQ) (ρ = −0.58). Additionally, the questions about ‘*understanding your back problem*’ from the BIPQ and the PEI-BP instruments were highly correlated (ρ = 0.60). A moderate correlation was seen between enablement and degree of control and not a high correlation as expected (ρ = 0.43). The expected moderate correlations were correctly predicted and indicated that a higher score regarding enablement to some extent correlates with better mental health (ρ = 0.50) and a less fear avoidant patient (ρ = −0.30). In contrast, the degree of enablement seems independent of the patients’ educational level (ρ = 0.13) and of the number of previous episodes of LBP (ρ = −0.04) as these hypotheses about low correlations were correctly predicted.

### Reliability

#### Internal consistency

Cronbach’s alpha for PEI-BP was 0.88. The alphas of the individual items ranged from 0.85 to 0.87 indicating no reason to perform item reduction.

#### Test–retest reliability

The mean response time between the test and retest was 4.9 days (SD =  ± 1.1). ICC was 0.74 (95% CI 0.54, 0.86) among the patients.

#### Measurement error

**Fig. 4:**
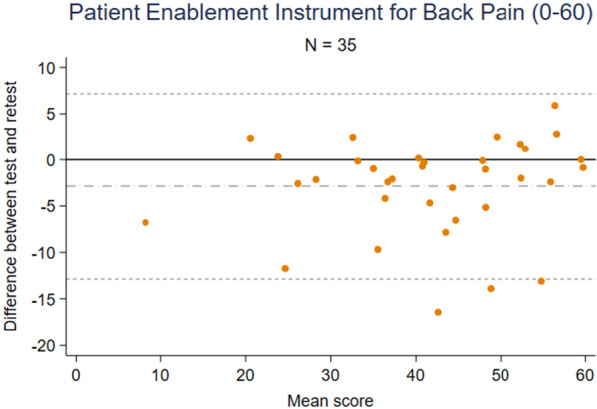
Bland and Altman plot showing the difference between the test and retest responses on the Patient Enablement Instrument for Back Pain, N = 35

The average difference in scores between test and retest was -2.9, which means that participants systematically rated a 2.9 lower score at retest (Fig. 4). The limits of agreement were -12.9 and 7.2 on a scale ranging from 0 to 60 points (SDC = 10.1).

The data were spread uniformly in the Bland and Altman plot (Fig. 4) after deleting two outliers (observations) from the analysis of limits of agreement as their difference of test and retest scores were more than double of the standard deviation.

### Responsiveness

#### Construct responsiveness

The proportion of correctly predicted hypotheses was 83% (5/6) (Table 5).

**Fig. 5:**
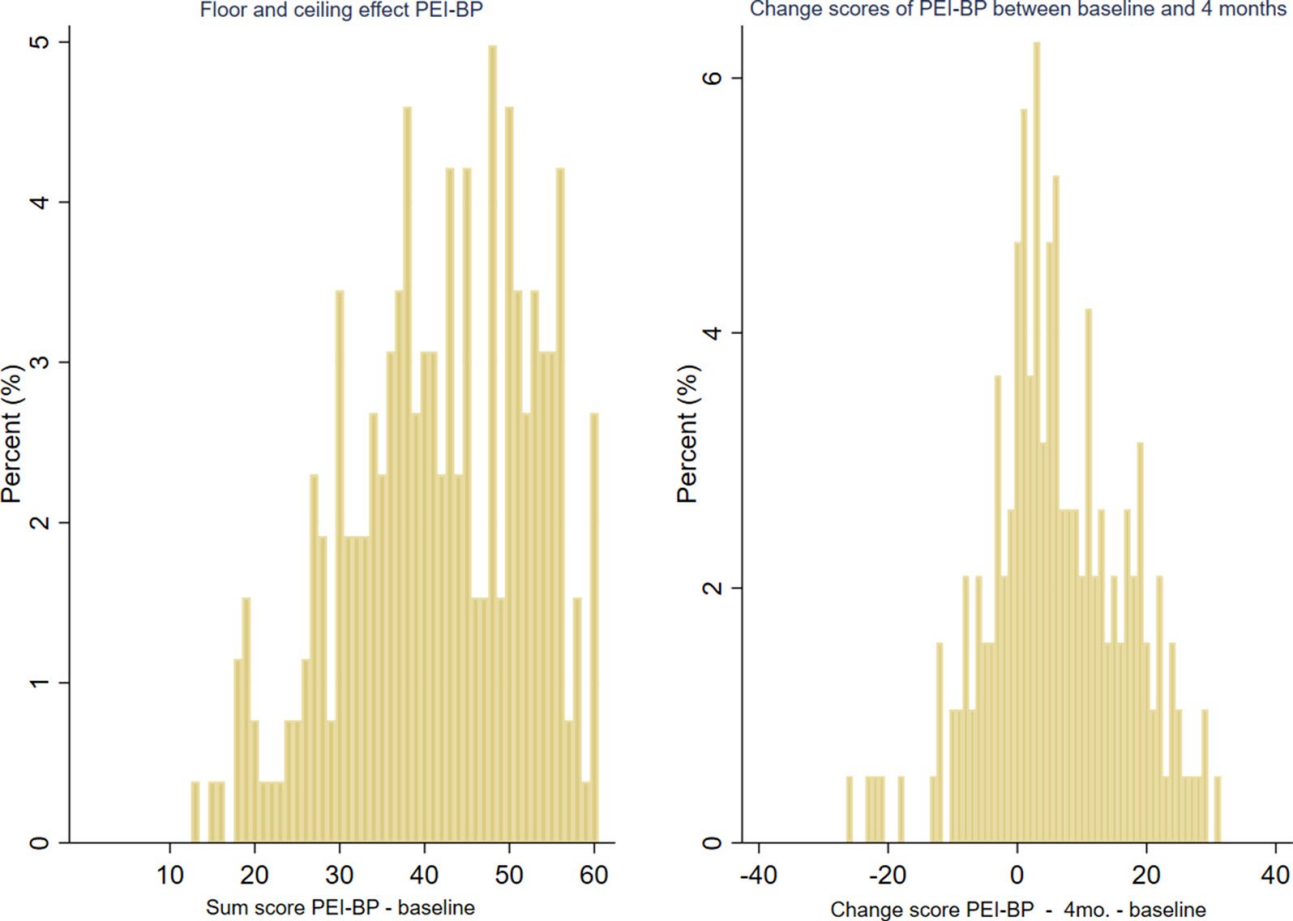
The distribution of baseline sum scores and change scores between baseline and 4 mo. follow-up on the Patient Enablement Instrument for Back Pain (PEI-BP) based on 261 and 191 participants, respectively, with non-specific low back pain. A higher score indicates higher enablement

The results indicated that a change to a higher degree of enablement highly correlates with a change to a lower degree of disability (ρ = −0.56), lower degree of illness beliefs (ρ = −0.54) and a higher degree of feeling of control (ρ = 0.55). When comparing the change score for the items about *‘understanding your back pain/ back problem’* from the BIPQ and the PEI-BP instruments it showed a moderate positive correlation (ρ = 0.46), and not high correlation as expected. The expected moderate correlations were correctly predicted and indicated that a change to a higher degree of enablement to some extent correlates with a change to better mental health (ρ = 0.41) and a change to a less fear avoidant patient (ρ = −0.43) after an intervention. An overview of the change scores from baseline to 4 months is shown in Fig. 5 (PEI-BP sum score) and in Fig. A1 of Additional file 2 (for each item of the PEI-BP).

### Floor and ceiling effects

A floor and ceiling effect of 0% and 29.9%, respectively, were found using the scale width method (Table 6). Especially item 6 (manage your life independently) contributed to the ceiling effect as 43% of the baseline scores had a maximum score of 10 (Fig. 5 + Table 3).

**Table 6:** Floor and ceiling effects of the Patient Enablement Instrument for Back pain based on 261 participants with non-specific low back pain

	Scale range	Classical method		Scale width method	
		Floor effect (%)	Ceiling effect (%)	Floor effect (%)	Ceiling effect (%)
PEI-BP sum score	0–60	0.0	2.7	0	29.9

## Discussion

We have described the development and measurement properties of the PEI-BP that focusses on the enablement of people to manage their back pain. Overall, the results showed satisfactory construct validity (structural validity and hypothesis testing) and reliability (internal consistency, test–retest reliability and measurement error) of the PEI-BP for use in research. The PEI-BP seemed responsive with higher PEI-BP scores relating to less disability and a less threatening view of back pain and to overall positive changes in health between baseline and follow-up. The skewed distribution towards the high scores indicates a problematic ceiling effect as the intention is to use PEI-BP to measure change over time. As the scale range on the original PEI was 0–2 compared to our 0–10, this issue has to our knowledge not been identified previously for individual items.

### Content validity

The questionnaire showed acceptable content validity as the participants in the content validity cohort generally found the questionnaire meaningful. However, they did find that item 3 overlapped with other items and therefore caused some uncertainty regarding its comprehensibility. A rewording of item 3 or addition of clarifications to each of the items, might be a way of improving the content validity of the PEI-BP, however based on the quantitative analysis, the overlap between item 3 and other items were not apparent. For example, we did not find an increased number of missing responses for item 3, nor did we find less impact on other clinimetric properties and therefore a rewording of item 3 is not of high priority.

### Construct validity

Exploratory factor analysis demonstrated a 1-factor model suggesting that the instrument is unidimensional and indeed measures the construct of patient enablement. This result is supported by the majority (3/4) of previous studies using factor analysis on the original PEI which have also shown unidimensionality [10, 11, 13, 16].

The PEI-BP also seem to be valid in terms of measuring what it purports to measure when compared to instruments measuring *back-related disability* (ODI) and *back pain beliefs* (BIPQ)*.* The PEI-BP seems related to the *feeling of control*, but not as highly as expected. This indicates a difference from the construct measured by BIPQ-control (“*how much control do you feel you have over your back pain”*) and maybe because PEI-BP covers a broader aspect than control as purely control of symptoms. As expected, PEI-BP seems moderately related to *physical activity* (FABQ) and *Mental health* (SF-36) and therefore, they are related but not measuring the same construct. Generally, these results are comparable to previous studies which have used the original PEI-BP such as Enthoven et al. who found a fair to moderate relationship with less disability and better mental and general health for a cohort of patients with chronic musculoskeletal pain [13]. Likewise, Haughney et al. found an association between the PEI and improved quality of life for patients with asthma using a modified version of the PEI [14]. However, a randomised controlled trial on people with chronic back pain by Eardley et al. did not show an association between enablement and disability at 5 weeks, potentially indicating a less robust relationship between the PEI and disability (Roland Morris Disability Questionnaire) [49]. Lastly with regards to the included hypotheses, we did not find that enablement was related to patients’ educational level or the number of previous episodes of LBP. In contrast, Ozvacić Adzić et al. found that the enablement score increased with higher educational level [15].

### Reliability

The internal consistency of the PEI-BP was satisfactory (α = 0.88) [24]. All the items seem to be interrelated and to measure the same construct. The result is comparable to studies on the original PEI within primary care with Cronbach’s alpha values between 0.84 and 0.93 [7, 10, 12]. The SDC at 10.1 points illustrates that a patient would have to change more than 1/6 of the scale range for it to be a true change.

### Responsiveness

The PEI-BP was responsive when compared to commonly used instruments for patients with LBP (*disability* (ODI) [*r* = −0.56] and *back pain beliefs* (BIPQ) [*r* = −0.54]). One hypothesis comparing *‘understanding your back pain / back problem’* (Table 5) did reach the pre-defined level of correlation (r = 46 versus r > 0.5), nevertheless, we believe that the PEI-BP can be used longitudinally to measure change over time and as an outcome measure.

### Floor and ceiling effects

The results of our hypothesis testing showed a high correlation between a low disability (ODI) score and a high enablement (PEI-BP) score, and as the average baseline ODI score was quite low in our population, this might explain part of the high PEI-BP score at baseline. The potential ceiling effect should be addressed in further development of the PEI-BP. From the item analysis, item 6 (manage your life independently) and item 1 (handle your everyday life) particularly seem to add to this effect. The original PEI was not developed to measure change over time and the scale range was smaller, however, Remelhe et al. did also identify a tendency for ceiling effects when using the original scale (0–2) [11]. Because our population scores high on especially the ability to manage and handle life already at the first response, one way of addressing the ceiling effect could be to make the anchors more extreme, i.e. changing from ‘to a very high degree’ to ‘an extreme degree’ or ‘completely’. Potentially, removing item 6 in populations with minor activity limitations, might have a positive impact on the identified ceiling effect. Importantly, floor- and ceiling effects are sensitive to the population being studied, so this might not be a problem if the instrument is used in other more severely affected LBP patients.

### Strengths and weaknesses

The relatively large number of participants is a strength in the context of the planned statistical analysis, although the number of included participants in the test–retest cohort preferable should have been higher and therefore, the results based on those data should be interpreted with caution.

The use of 2 cohorts for different analysis is also a potential weakness, however the analyses revealed no statistical baseline differences between the two cohorts.

The majority of participants were females, which might reduce the generalisability of the study [8, 50–52], however, other studies have not found a significant difference in score with regards to sex [16, 53]. Additionally, the mentioned studies were based on PEI and not PEI-BP and therefore, interpretation and comparison should be made with caution.

## Conclusions

Based on the Patient Enablement Instrument, we developed the Patient Enablement Instrument for Back Pain, PEI-BP. The PEI-BP has acceptable content validity, construct validity, reliability (internal consistency, test–retest reliability and measurement error) and responsiveness. Thus, the PEI-BP can be considered a valid and reliable tool to measure enablement in people seeking care for non-specific LBP in research settings. Further testing of the PEI-BP in populations with more severe LBP is recommended.

## Supplementary Information

**Additional file 1:** A Danish, English and Swedish version of the Patient Enablement Instrument for Back Pain questionnaire (PEI-BP).

**Additional file 2:** A figure showing the change scores from baseline to 4 months of the six individual items of the Patient Enablement Instrument for Back Pain.

## Data Availability

The datasets used and analysed during the current study are available from the corresponding author on reasonable request.

## Abbreviations

BIPQ: Brief illness perception questionnaire
CI: Confidence intervals
COSMIN: COnsensus-based Standards for the selection of health Measurement INstruments
EFA: Explorative factor analysis
FABQ: Fear-avoidance beliefs questionnaire
GLA:D®: Good life with osteoarthritis in Denmark
LBP: Low back pain
ODI: Oswestry disability index
OPEN: Open Patient data Explorative Network
PEI: Patient enablement instrument
PEI-BP: Patient enablement instrument for back pain
SBT: STarT back tool
SDC: Smallest detectable change
SF-36: Short form 36
