# Supplementary material for: The patient enablement instrument for back pain: reliability, content validity, construct validity and responsiveness
Source: Health Qual Life Outcomes. 2021 Apr 9;19:116. doi: 10.1186/s12955-021-01758-0 (PMC8033700; doi:10.1186/s12955-021-01758-0)
Supplement: Supplementary file 1 — Additional file 1: A Danish, English and Swedish version of the Patient Enablement Instrument for Back Pain questionnaire (PEI-BP). [file 12955_2021_1758_MOESM1_ESM.pdf]

Additional file 1. A Danish, English and Swedish version of the Patient Enablement Instrument for Back Pain questionnaire (PEI-BP)

Danish version of the PEI-BP questionnaire

Patient Enablement Instrument - rygsmerter

Rygsmerter og din hverdag

**Når du tænker tilbage på den seneste uge – i hvilken grad kunne du:**	I meget lav grad									I meget høj grad
**håndtere din hverdag**	0	1	2	3	4	5	6	7	8	9	10
**forstå dit rygproblem**	0	1	2	3	4	5	6	7	8	9	10
**klare dit rygproblem**	0	1	2	3	4	5	6	7	8	9	10
**holde din ryg sund**	0	1	2	3	4	5	6	7	8	9	10
**føle dig tryg ved dit helbred**	0	1	2	3	4	5	6	7	8	9	10
**klare dig selv**	0	1	2	3	4	5	6	7	8	9	10

The Patient Enablement Instrument for Back Pain (PEI-BP)

Back pain and your everyday life

**When you look back at the past week – to which degree were you able to:**	To a very low degree										To a very high degree
**handle your everyday life**	0	1	2	3	4	5	6	7	8	9	10
**understand your back problem**	0	1	2	3	4	5	6	7	8	9	10
**manage your back problem**	0	1	2	3	4	5	6	7	8	9	10
**keep your back in good health**	0	1	2	3	4	5	6	7	8	9	10
**feel confident with your health**	0	1	2	3	4	5	6	7	8	9	10
**manage your life independently**	0	1	2	3	4	5	6	7	8	9	10

Patient Enablement Instrument - ryggsmärta

När du tänker tillbaka på senaste veckan
– till vilken grad kan du
(Ringa in den siffra som bäst överensstämmer med din åsikt)

	I mycket låg grad										I mycket hög grad
**Hantera ditt liv**	0	1	2	3	4	5	6	7	8	9	10
**förstå dina (rygg)besvär**	0	1	2	3	4	5	6	7	8	9	10
**hantera dina (rygg)besvär**	0	1	2	3	4	5	6	7	8	9	10
**hålla dig själv frisk/vid god hälsa**	0	1	2	3	4	5	6	7	8	9	10
**känna dig trygg beträffande din hälsa**	0	1	2	3	4	5	6	7	8	9	10
**hjälpa dig själv**	0	1	2	3	4	5	6	7	8	9	10

© 2000, Öberg, Enthoven, Abbott, Linköping University, Sweden.
