# Supplementary material for: The patient enablement instrument for back pain: reliability, content validity, construct validity and responsiveness
Source: Health Qual Life Outcomes. 2021 Apr 9;19:116. doi: 10.1186/s12955-021-01758-0 (PMC8033700; doi:10.1186/s12955-021-01758-0)
Supplement: Supplementary file 2 — Additional file 2: A figure showing the change scores from baseline to 4 months of the six individual items of the Patient Enablement Instrument for Back Pain. [file 12955_2021_1758_MOESM2_ESM.pdf]

Additional file 2. A figure showing the change scores from baseline to 4 months of the six individual items of the Patient Enablement Instrument for Back Pain

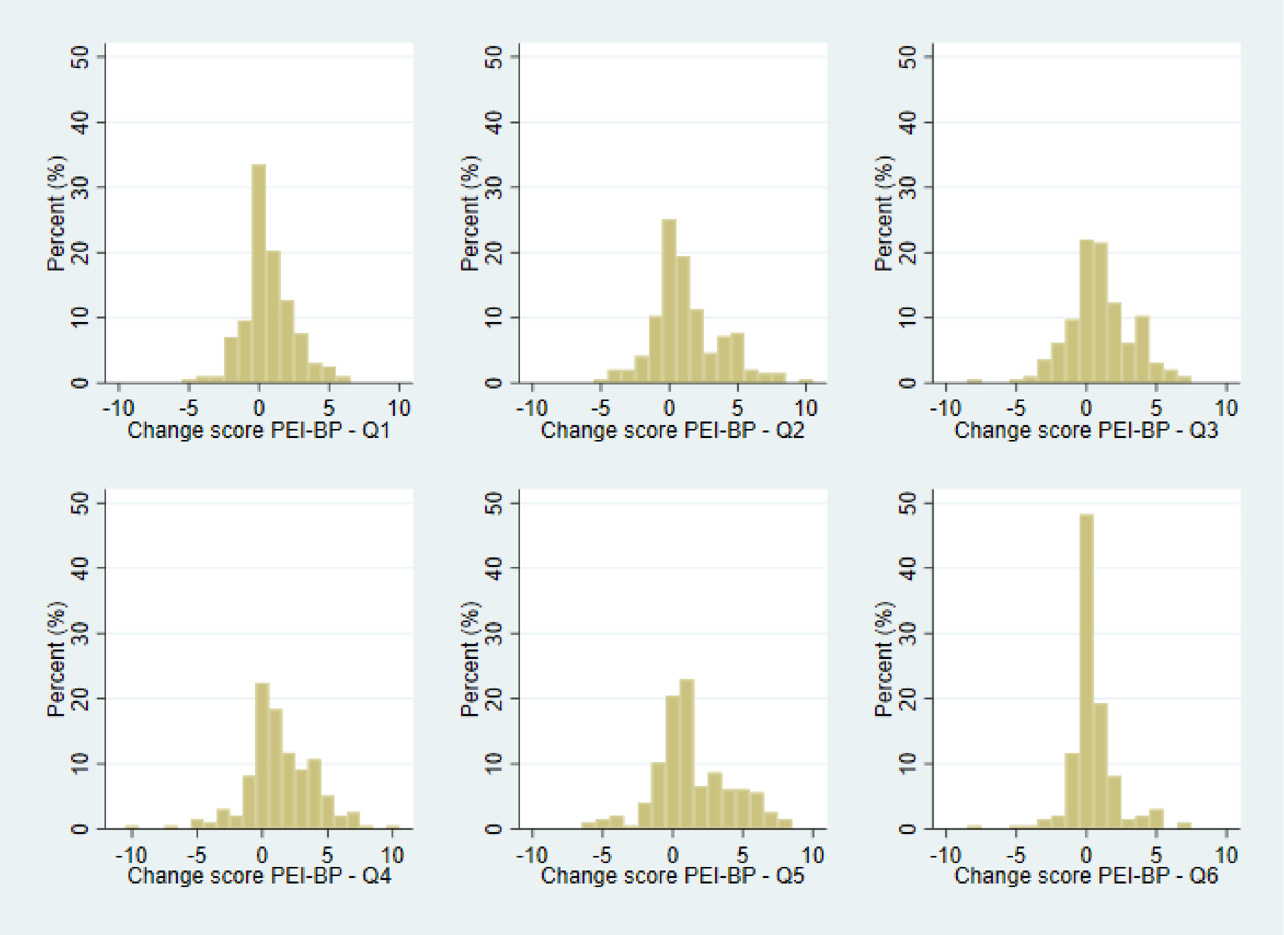

Fig. A1. Change scores from baseline to 4 months on the six individual items of the Patient Enablement Instrument for Back Pain. The number of respondents ranged from 195 to 198.
